# Meta-Analysis of Randomized Trials: Efficacy and Safety of Colchicine for Secondary Prevention of Cardiovascular Disease

**DOI:** 10.1155/2024/8646351

**Published:** 2024-03-12

**Authors:** Elie Akl, Nazanin Sahami, Christopher Labos, Jacques Genest, Ali Zgheib, Nicolo Piazza, Sanjit Jolly

**Affiliations:** ^1^McGill University, Montreal, Canada; ^2^McMaster University, Hamilton, Canada

## Abstract

**Background:**

Colchicine has shown potential cardioprotective effects owing to its broad anti-inflammatory properties. We performed a meta-analysis to assess its safety and efficacy in secondary prevention in patients with established coronary artery disease (CAD).

**Methods:**

We searched Ovid Healthstar, MEDLINE, and Embase (inception to May 2022) for randomized controlled trials (RCTs) evaluating the cardiovascular effects of colchicine compared with placebo or usual care in patients with CAD. Study-level data on efficacy and safety outcomes were pooled using the Peto method. The primary outcome was the composite of cardiovascular (CV) death, myocardial infarction (MI), or stroke.

**Results:**

A total of 8 RCTs were included with a follow-up duration of ≥1 month, comprising a total of 12,151 patients. Compared with placebo or usual care, colchicine was associated with a significant risk reduction in the primary outcome (odds ratio (OR) 0.70, 95% CI 0.60 to 0.83, *P* < 0.0001; *I*^2^ = 52%). Risks of MI (OR 0.75, 95% CI 0.62 to 0.91, *P* = 0.003; *I*^2^ = 33%), stroke (OR 0.47, 95% CI 0.30 to 0.74, *P* = 0.001; *I*^2^ = 0%), and unplanned coronary revascularization (OR 0.67, 95% CI 0.55 to 0.82, *P* = 0.0001; *I*^2^ = 58%) were all reduced in the colchicine group. Rates of CV and all-cause mortality did not differ between the two groups, but there was an increase in noncardiac deaths with colchicine (OR 1.54, 95% CI 1.10 to 2.15, *P* = 0.01; *I*^2^ = 51%). The occurrence of all other adverse events was similar between the two groups, including GI reactions (OR 1.06, 95% CI 0.94 to 1.20, *P* = 0.35; *I*^2^ = 42%) and infections (OR 1.04, 95% CI 0.84 to 1.28, *P* = 0.74; *I*^2^ = 53%).

**Conclusions:**

Colchicine therapy may reduce the risk of future cardiovascular events in patients with established CAD; however, there remains a concern about non-CV mortality. Further trials are underway that will shed light on non-CV mortality and colchicine NCT03048825, and NCT02898610.

## 1. Introduction

### 1.1. Background and Rationale

Patients with established coronary artery disease (CAD) remain at high risk for recurrent cardiovascular events despite current evidence-based secondary prevention that includes antithrombotic and lipid therapies alongside lifestyle changes [1]. The residual incidence of such events is estimated at 3–5% per year in patients treated with guideline-directed medical therapy [2, 3]. Improvements to current treatment options are still needed.

Persistent subclinical coronary inflammation is perceived as a key driver of residual risk. A number of studies suggested that inflammatory biomarkers play a pivotal role in the development and progression of atherosclerosis [4, 5], and research has now progressed into clinical trials investigating whether specifically targeting inflammation prevents cardiovascular events [6]. Colchicine is a low-cost drug that has been used for many years for the treatment of inflammatory conditions such as gout, pericarditis, and familial Mediterranean fever (FMF). It interferes with several steps in the inflammatory process and has an antitubulin effect that inhibits neutrophil function. In trials of gout [7] and FMF [8], retrospective observations suggested a cardioprotective effect with continued use of colchicine [9]. More recently, large randomized controlled trials (RCTs) showed potential benefit with colchicine in patients with stable coronary disease [10] and post-ACS [11]. We therefore conducted a meta-analysis of RCTs to examine the efficacy and safety of colchicine for secondary prevention in patients with CAD.

## 2. Methods

### 2.1. Protocol and Registration

This meta-analysis was conducted in accordance with the Cochrane Handbook for Systematic Reviews and Interventions [12] and reported following the PRISMA checklist for meta-analysis in healthcare interventions [13]. A protocol specifying the objectives, inclusion criteria, and analysis methods was submitted to PROSPERO [14] on January 21, 2021, and registered with the number CRD42021227630.

### 2.2. Information Sources

We conducted systematic searches for relevant studies comparing colchicine versus placebo or usual care for secondary cardiovascular prevention up to May 30, 2022. This included searching Ovid Healthstar (from 1966), MEDLINE (from 1948), and Embase (from 1980) as well as the Cochrane Central Register of Controlled Trials. Reference lists of included studies, relevant articles, and related systematic reviews were also reviewed. To identify any ongoing or recently completed studies that have not been published, we searched conference abstracts from the American College of Cardiology (ACC), the American Heart Association (AHA), Transcatheter Therapeutics (TCT), and the European Society of Cardiology (ESC) from the last three years. Web-based registries were also searched, including ClinicalTrials.gov and PROSPERO to identify completed studies that have not been published.

A search strategy was created for Ovid Healthstar and modified for application to the other databases (Figure S1). The key concepts of the search strategy included the intervention (“colchicine”), disease/morbidity subject terms (“coronary artery disease,” “coronary heart disease,” “myocardial ischemia,” “cardiovascular diseases,” “acute coronary syndrome,” and “myocardial infarction”), and study type (randomized trials). The search was limited to the English language. Review articles, editorials, duplicates, and post hoc analyses of original RCTs were excluded.

### 2.3. Eligibility Criteria

To be included in this meta-analysis, all RCTs had to meet the following criteria: (1) studies that compared colchicine versus placebo or usual care for secondary cardiovascular prevention; (2) studies that reported at least one of the following outcomes: cardiovascular death, myocardial infarction (MI), and stroke; (3) colchicine was administered at any dose for a minimum of 30 days; and (4) follow-up duration was at least 30 days.

### 2.4. Outcome Measures and Certainty of Evidence

Information on study outcomes was abstracted for the longest available follow-up. The primary outcome was the composite of cardiovascular (CV) death, MI, or stroke. Secondary analyses were conducted on the individual components of the primary outcome, as well as all-cause mortality and unplanned coronary revascularization. Safety outcomes included noncardiovascular mortality, gastrointestinal events, infection, cancer, myalgia, and myelotoxicity. The Grading of Recommendations Assessment, Development, and Evaluation (GRADE) approach was used to evaluate the certainty of the evidence presented in this study [15]. The GRADE approach incorporates evaluations on trial risk of bias, inconsistency, indirectness, imprecision, and publication bias.

### 2.5. Study Selection, Data Extraction, and Bias Assessment

Study selection was performed by two independent review authors (E.A. and N.S.). Titles and abstracts were screened for eligibility followed by full-text review. Reasons for exclusion were documented. Disagreements in each stage were resolved by discussion and consensus. An electronic database was developed to document study characteristics and outcome data on the intent-to-treat population. One reviewer entered the data (E.A.), which was subsequently validated by a second reviewer (N.S.).

The risk of bias was assessed in the 5 domains as recommended in the Cochrane Handbook for Systematic Reviews of Interventions. These domains included risk of bias (1) arising from the randomization process; (2) due to deviations from the intended interventions; (3) related to missing outcome data; (4) in the measurement of the outcome; and (5) in the selection of the reported result (Figure S2).

### 2.6. Statistical Analysis

Peto odds ratios (ORs) were calculated to display dichotomous outcomes. Peto's fixed-effect method was used to calculate the pooled Peto ORs and corresponding 95% confidence intervals (CIs) when two or more studies provided combinable data. Heterogeneity was evaluated with the Cochrane *Q* test (significant at *P* < 0.10) and the Higgins *I*^2^ statistic. Published guidelines for low (*I*^2^=25–49%), moderate (*I*^2^=50–74%), and high (*I*^2^ ≥ 75%) heterogeneity were used [16]. Subgroup analyses for the primary outcome were performed according to (1) treatment duration (30 days, >30 days), (2) colchicine dosage (<1 mg/d, ≥1 mg/d), and (3) CAD phenotype (stable CAD vs ACS presentation). We expected a greater benefit as treatment duration increased and with a larger dosage of colchicine. We also expected heterogeneity by baseline CAD phenotype since the risk for cardiovascular events would differ. Publication bias was evaluated using the inverted funnel plot techniques (significant at *P* < 0.10) [17, 18]. As a sensitivity analysis, we also performed a Bayesian meta-analysis using a noninformative prior distribution. This allowed us to calculate the posterior probability and estimate the probability of any benefit or of a clinically significant benefit for each of the specified endpoints. All analyses were performed using Review Manager (RevMan), version 5.3 (Nordic Cochrane Center, Cochrane Collaboration, Copenhagen), and STATA, version 17 (StataCorp LP, College Station, Texas, USA).

## 3. Results

### 3.1. Search and Selection of Studies

**Figure 1:**
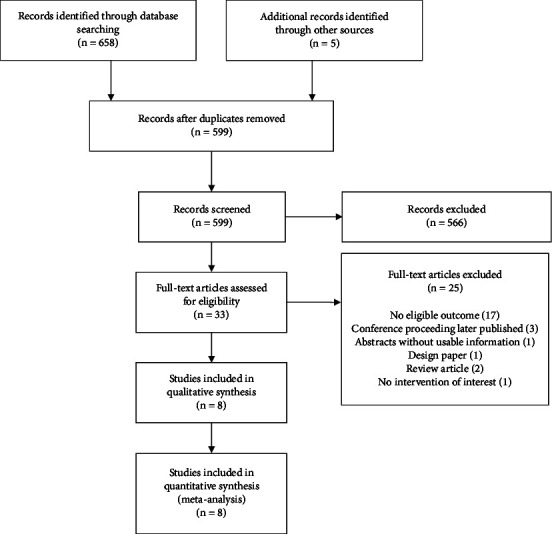
PRISMA flow diagram.

A total of 599 abstracts were retrieved from electronic databases and hand-searching conference proceedings and reference lists. Of the 33 studies selected for full-text review, 25 were eliminated after applying the eligibility criteria (Figure 1).

### 3.2. Study Characteristics and Risk of Bias within Studies

**Figure 2:**
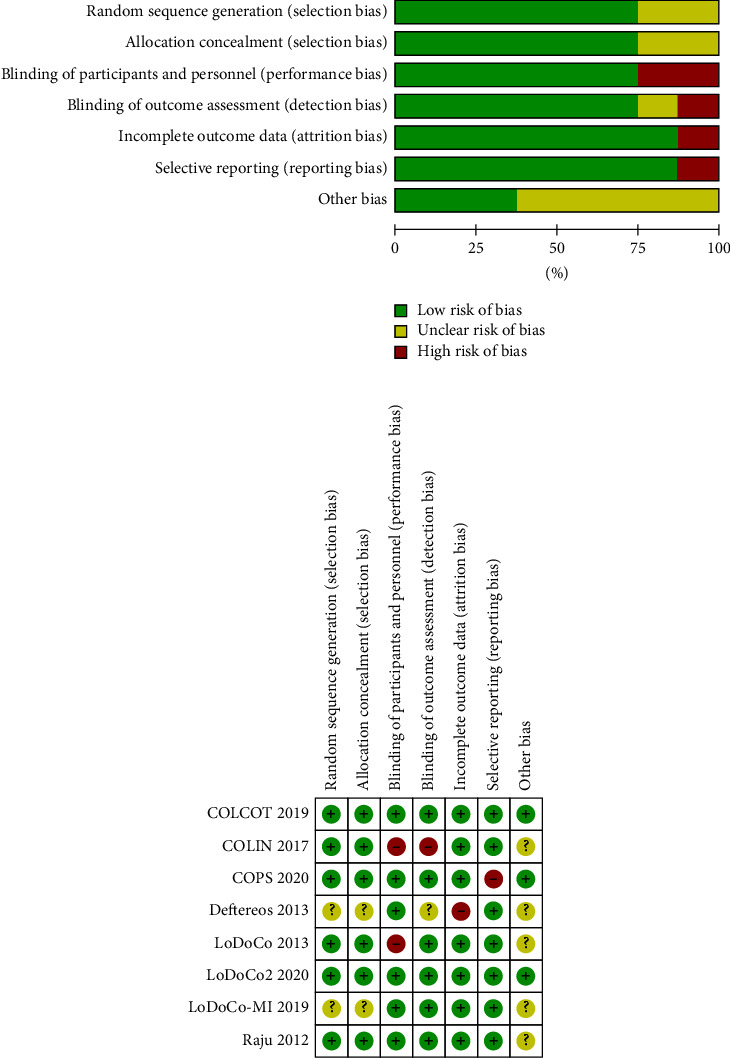
Risk of bias graph and summary. (a) Risk of bias graph: review authors' judgments about each risk of bias item presented as percentages across all included studies. (b) Risk of bias summary: review authors 'judgment about each risk of bias item for each included study.

Study characteristics for each RCT are listed in Table 1. Of the 8 included RCTs (*N* = 12,151), 6 involved patients with ACS [11, 19–23] and 2 exclusively enrolled patients with clinically stable CAD [10, 24]. The majority of trials used a colchicine dose of 0.5 mg daily. Three RCTs had a follow-up duration of 1 month [19–21], one RCT had a follow-up duration of 6 months [23], and all 4 other trials had follow-up durations of at least 1 year [10, 11, 22, 24]. Figures 2(a) and 2(b) summarize the risk of bias for the included RCTs and individual assessments. The LoDoCo [24] and COLIN [20] trials were the only open-label RCTs comparing colchicine to usual care, but the selection bias was minimized in both trials through allocation concealment, and detection bias was reduced in LoDoCo through blinded outcome assessment.

### 3.3. Patient Characteristics

Patient characteristics are listed in Table 2. The mean age of patients ranged between 57 and 67. Over 75% of enrolled patients were male in all trials. The prevalence of hypertension, diabetes, and smoking was well balanced between the intervention and control arms of the studies. Use of secondary prevention drugs was high across trials, and more than 90% of patients were on concomitant statin therapy.

### 3.4. Summary of Findings

Table S1 summarizes the findings for the primary and secondary outcomes including the GRADE quality assessment.

### 3.5. Efficacy Outcomes

**Figure 3:**
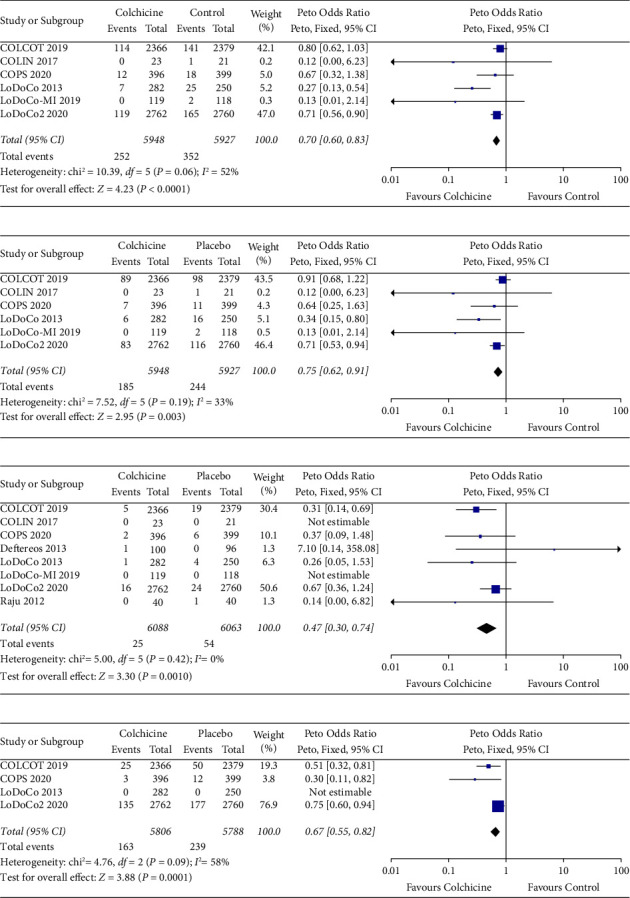
Outcomes. (a) CV death, MI, or stroke. (b) Myocardial infarction. (c) Stroke. (d) Unplanned coronary revascularization.

**Figure 4:**
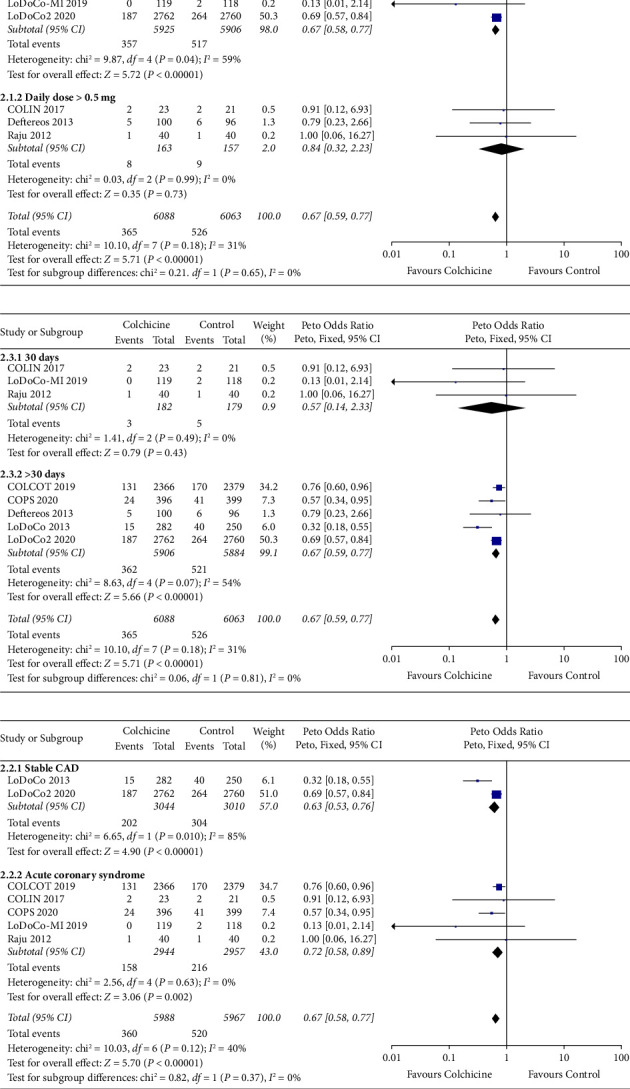
Subgroup analyses. (a) Colchicine dose. (b) Treatment duration. (c) CAD phenotype.

Compared with placebo or usual care, the addition of colchicine to standard medical therapy in patients with CAD was associated with a significant reduction in the primary outcome of CV death, MI, or stroke (OR 0.70, 95% CI 0.60 to 0.83, *P* < 0.0001; *I*^2^=52%) (Figure 3). There was also a reduction in MI (OR 0.75, 95% CI 0.62 to 0.91, *P*=0.003; *I*^2^=33%), stroke (OR 0.47, 95% CI 0.30 to 0.74, *P*=0.001; *I*^2^=0%), and unplanned coronary revascularization (OR 0.67, 95% CI 0.55 to 0.82, *P*=0.0001; *I*^2^=58%). The reduction in the primary outcome was consistent between subsets of trials that differed according to treatment duration (*P* for interaction = 0.81), colchicine dose (*P* for interaction = 0.65), and CAD phenotype at baseline (*P* for interaction = 0.37) (Figure 4). Excluding one study at a time in a “one-out” sensitivity analysis did not neutralize the pooled Peto OR of the primary outcome. Excluding results from the LoDoCo and COLIN open-label trials did not alter primary outcome results, although the magnitude of effect and heterogeneity (*I*^2^) between studies were decreased (Figure S3). The Bayesian posterior probability of colchicine reducing the primary endpoint was 99%.

### 3.6. Mortality and Other Adverse Events

**Figure 5:**
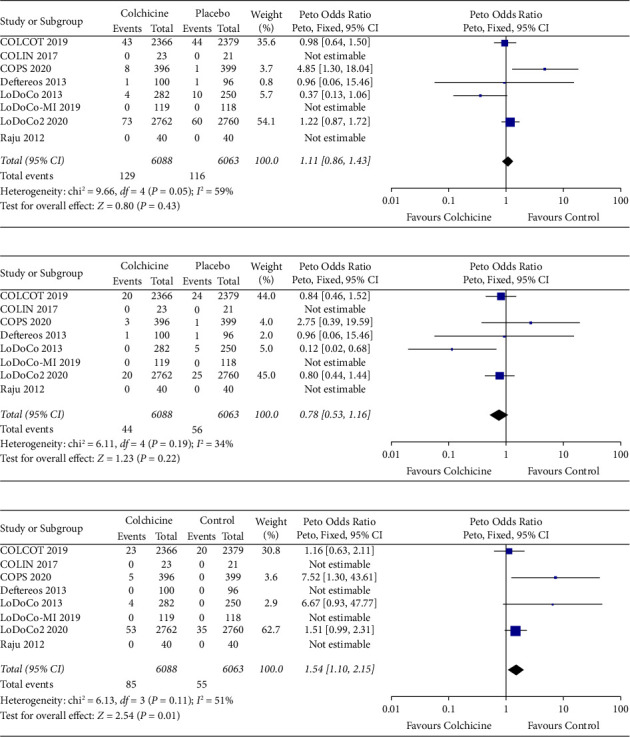
Mortality. (a) All-cause mortality. (b) CV mortality. (c) Non-CV mortality.

All-cause mortality was similar between the two groups (OR 1.11, 95% CI 0.86 to 1.43, *P*=0.43; *I*^2^=59%). The Bayesian posterior probability for an increase in all-cause mortality was 67.47%. There was no significant decrease in cardiovascular mortality with colchicine therapy (OR 0.78, 95% CI 0.53 to 1.16, *P*=0.22; *I*^2^=39%). However, noncardiovascular mortality was significantly increased with colchicine (OR 1.54, 95% CI 1.10 to 2.15, *P*=0.01; *I*^2^=51%) (Figure 5). The Bayesian posterior probability for any increase in noncardiovascular mortality was 96.75%. The probability for a greater than 5% increase on the relative scale was 95.29%.

Overall, colchicine was not associated with a significant increase in GI adverse events (OR 1.06, 95% CI 0.94 to 1.20, *P*=0.35; *I*^2^=42%) or infections (OR 1.04, 95% CI 0.84 to 1.28, *P*=0.74; *I*^2^=53%) compared with placebo or usual care. The Bayesian posterior probability for both outcomes was 86.36% and 64.26%, respectively. Risks of treatment discontinuation, cancer, myalgia, and myelotoxicity were also similar between the two groups (Table 3).

### 3.7. Risk of Publication Bias

Visual inspection of the funnel plots did not show asymmetry and suggested no significant risk of publication bias (Figure S4).

## 4. Discussion

The present meta-analysis included eight RCTs of patients receiving colchicine for secondary prevention of cardiovascular diseases. Colchicine was associated with a significant risk reduction in the primary outcome of CV death, MI, or stroke as compared with placebo or usual care. There was also a significant reduction in the individual outcomes of MI, stroke, and unplanned coronary revascularization but not CV death. These findings were consistent between subsets of trials that differed according to CAD phenotype at baseline (ACS vs stable CAD). However, there was an increase in noncardiovascular mortality in the colchicine group. This finding is uncertain given the significant heterogeneity. Colchicine did not increase the risk of all other major adverse events, including gastrointestinal reactions and infections.

The plausibility of reduction of cardiovascular events by targeting coronary inflammation is well supported in the literature. The role of proteolytic enzymes released as part of the chronic inflammatory process leading to atherosclerotic plaque erosion or rupture leading to recurrent coronary events has been well described [25]. Statins have been shown to reduce cardiovascular events through the reduction of inflammation in addition to their low-density lipoprotein (LDL)-lowering properties [26]. In the CANTOS trial, canakinumab significantly reduced the rate of recurrent cardiovascular events compared with placebo by targeting the interleukin-1*β* innate immunity pathway in patients with previous MI and elevated high-sensitivity C-reactive protein levels [6]. It is now appreciated that extracellular cholesterol crystals trigger inflammatory processes upstream and downstream of the interleukin-1*β* pathway, suggesting that the broad anti-inflammatory properties of colchicine may be required to adequately tackle the atherosclerotic inflammatory process [27]. The mechanisms of action of colchicine are complex and include microtubule assembly, inflammasome activation, inflammatory cell chemotaxis, leukotriene and cytokine generation, and phagocytosis. Recently, colchicine has been found to attenuate the nucleotide-binding oligomerization domain-, leucine-rich repeat-, and pyrin domain-containing protein-3 (NLRP3) inflammasome-mediated crystal-induced inflammation [9]. A proteomic analysis of the LoDoCo-2 trial performed after 30 days of colchicine treatment revealed a reduction of interleukin IL-18, IL-1 receptor antagonist, and IL-6, consistent with an attenuation of the NLRP3 inflammasome pathway. There was also a reduction of the upstream NF-*κ*B essential modulator, required in NLRP3 activation [28]. In a large meta-analysis investigating the effects of colchicine on inflammatory markers in patients with CAD across 11 clinical trials, colchicine led to a significant reduction in hs-CRP (weighted mean differences (WMDs), −0.81 mg/L; 95% confidence interval (CI), −1.34 to 0.28 mg/L; *P*=0.003) and IL-6 levels (WMD, −1.28 pg/mL; 95% CI, −2.35 to −0.21 pg/mL; *P*=0.02) compared with placebo [29]. From a clinical standpoint, colchicine is inexpensive and taken orally. On the other hand, widespread use of canakinumab has been limited due to increased risk of fatal infections, elevated cost, and inconvenience related to subcutaneous administration.

Although the risk of ischemic cardiovascular events in colchicine-treated patients was significantly reduced, it did not translate into an overall survival benefit, and noncardiovascular mortality was increased with colchicine therapy. These findings, along with the CANTOS trial results, perhaps indicate that immunomodulating therapy in patients with established CAD may bring about cardiovascular benefits at a cost of noncardiac deaths. Future research should focus on evaluating the safety of colchicine in this patient population, especially its impact on noncardiac mortality and serious infections. Such research includes the Colchicine and Spironolactone in Patients With MI/SYNERGY Stent Registry (CLEAR SYNERGY) trial, which uses a 2 × 2 factorial design to examine the effect of colchicine and spironolactone in patients presenting within STEMI treated with primary PCI or high-risk non-ST-segment elevation myocardial infarction (ClinicalTrials.gov identifier: NCT03048825).

The risk reduction in stroke with colchicine therapy was substantial and reinforced the role of colchicine as a treatment of inflammation-related atherothrombosis. It may be especially relevant to neurologists and other healthcare professionals involved in secondary stroke prevention. The colchicine for prevention of vascular inflammation in noncardio embolic stroke (CONVINCE) will further inform us on the role of low-dose colchicine for secondary prevention of recurrent stroke and major vascular events in patients who have already suffered a nonembolic ischemic stroke or transient ischemic attack (ClinicalTrials.gov identifier: NCT02898610).

The lack of difference between groups for myalgia is especially reassuring given some existing concerns about increasing the risk of drug-induced myopathy when coprescribing statins and colchicine. Over 94% of patients in both groups were on statin therapy and yet such phenomenon was not observed.

Overall, our results are consistent with previous meta-analyses with smaller sample sizes [30, 31]. In a meta-analysis of four RCTs in which endpoints were harmonized across trials, treatment with colchicine was associated with a 32% reduction in the incidence of major cardiovascular events, but a trend towards increased noncardiovascular mortality was observed (pooled RR 1.38 (95% CI 0.99 to 1.93); *I*^2^=0.0%) [31]. Similarly, in a meta-analysis by the Colchicine Cardiovascular Trialists Collaboration, low-dose colchicine reduced the risk of MACE by 25% (RR 0.78, 95% CI 0.64 to 0.94; *P*=0.010) and there was no difference in all-cause death, with fewer cardiac deaths (RR 0.82, 95% CI 0.55 to 1.23; *P*=0.34) counterbalanced by a trend for more noncardiovascular deaths (RR 1.38, 95% CI 0.99 to 1.92; *P*=0.060) [32]. Compared with these two meta-analyses, ours included a larger number of patients and events and showed a statistically significant increase in non-CV death with colchicine. This finding remains uncertain given the significant heterogeneity, the fact that four of the trials were not evaluable due to lack of reported data and unexplained as rates of cancer and infections were not increased in the colchicine group.

### 4.1. Exploration of Heterogeneity

With the exclusion of the two open-label trials, a significant drop in heterogeneity was observed for the primary outcome of CV death, stroke, or MI (*I*^2^=0.0%) and its individual components of CV death (*I*^2^=0.0%), stroke (*I*^2^=12%), and MI (*I*^2^=7.0%) (Figure S5). This was not observed for non-CV mortality, so our findings related to this outcome should still be interpreted with caution. Also, our data do not provide sufficient evidence to determine clinical features that identify patients with CAD that are most likely to benefit or be harmed from colchicine therapy, such as patient age, comorbidities, and duration of therapeutic time window. The other major determinants of the heterogeneity observed in this analysis may remain uncertain until patient-level data analysis is performed.

### 4.2. Limitations

This meta-analysis does have limitations. First, we did not have access to individual patient data from the trials which would have allowed detailed analysis of subgroups. Second, our study may be underpowered to detect any potential difference in rare events such as cardiovascular death. Indeed, <1% of patients in both the colchicine and control groups in this meta-analysis died from cardiovascular causes. Third, the inclusion of the LoDoCo and COLIN trials may have introduced randomization bias due to their open-label design. However, bias was likely reduced thanks to allocation concealment in both trials. Furthermore, excluding these two trials did not alter our primary outcome results as shown in the sensitivity analysis (Figure S3). Fourth, duration of follow-up exceeded two years in only two trials (*n* = 6,054), preventing us from drawing any firm conclusion about the safety and efficacy of colchicine for secondary cardiovascular prevention on very long-term follow-up. Three trials (*n* = 363) had a short 30-day course of colchicine therapy, which poses challenges to interpretation of mortality outcomes. Finally, our meta-analysis was underpowered to detect the interaction between colchicine dose or duration and the beneficial effect of colchicine over placebo.

## 5. Conclusion

In randomized trials of patients receiving colchicine for secondary prevention of cardiovascular disease, colchicine led to a significant risk reduction in the composite outcome of CV death, MI, or stroke. However, there remains a concern for noncardiovascular mortality with colchicine in the CAD population. Therefore, although these findings may support the concept of targeting inflammation in atherosclerosis, further clinical research is required before colchicine can be safely administered routinely for secondary cardiovascular prevention.

## Figures and Tables

**Table 1:** Study characteristics.

Study	Raju et al.	Deftereos et al.	LoDoCo	COLIN	LoDoCo-MI	COLCOT	LoDoCo-2	COPS
Countries	Canada	Greece	Australia	France	Australia	12 countries	Australia and Netherlands	Australia
Total no. of Pts	82	222	532	44	237	4745	5522	795
Year	2012	2013	2013	2017	2019	2019	2020	2020
Population	ACS or acute ischemic stroke	Diabetes, age 40–80, undergoing PCI with a BMS	Clinically stable CAD for ≥6 months	STEMI	MI	MI	Chronic CAD	ACS
Colchicine dose	1 mg once daily	0.5 mg twice daily	0.5 mg once daily	1 mg once daily	0.5 mg daily	0.5 mg daily	0.5 mg daily	0.5 mg twice daily for 1 month, followed by 0.5 mg once daily for 11 months
Comparator	Placebo	Placebo	Usual care	Usual care	Placebo	Placebo	Placebo	Placebo
Duration of therapy (months)	1	6	24	1	1	19.6	Not specified	12
Follow-up (months)	1	6	36	1	1	22.6	28.6	13.3
Primary outcome	hs-CRP level	In-stent restenosis	ACS, OHCA, or noncardioembolic ischemic stroke	CRP peak value during index hospitalization	hs-CRP level ≥2 mg/L	CV death, resuscitated cardiac arrest, MI, stroke, or urgent hospitalization for angina requiring revascularization	CV death, MI, ischemic stroke, or ischemia-driven coronary revascularization	All-cause mortality, ACS, unplanned urgent revascularization, or noncardioembolic ischemic stroke
Source of funding	Academic	Not specified	Academic	None	Academic	Academic	Academic	Academic

ACS: acute coronary syndrome; BMS: bare metal stent; CAD: coronary artery disease; CRP: C-reactive protein; CV death: cardiovascular death; hs-CRP: high-sensitivity C-reactive protein; MACE: major adverse cardiovascular events; MI: myocardial infarction; PCI: percutaneous coronary intervention; STEMI: ST-segment elevation myocardial infarction.

**Table 2:** Patient characteristics at baseline.

Study	Raju et al.		Deftereos et al.		LoDoCo		COLIN		LoDoCo-MI		COLCOT		LoDoCo-2		COPS	
	Colchicine	Placebo	Colchicine	Placebo	Colchicine	Placebo	Colchicine	Placebo	Colchicine	Control	Colchicine	Placebo	Colchicine	Placebo	Colchicine	Nonexposed
Number of patients	40	40	100	96	282	250	23	21	119	118	2366	2379	2762	2760	396	399
Mean age (y)	57.2	57.2	63.7	63.5	66	67	60.1	59.7	61	61	60.6	60.5	65.8	65.9	59.7	60.0
Male (%)	85	93	63	68	89	89	82.5	76.2	75	79	80.1	81.6	83.5	85.9	81	78
Diabetes (%)	18	15	100	100	33	28	13.0	14.3	23	21	19.5	20.9	17.8	18.7	19	19
Hypertension (%)	48	38	48	49	—	—	39.1	47.6	54	41	50.1	52	51.4	50.3	51	50
Smoker (%)	45	43	36	40	4	6	73.9	66.7	65	57	29.9	29.8	11.5	12.0	32	37
Medications (%) Aspirin	100	100	—	—	93^1^	94^1^	-^2^	-^2^	99	100	98.6	98.9	66.9	67.1	99	98
Statin	100	95	—	—	96	94	—	—	97	100	98.9	99.1	93.9	94.0	98	99
ACEI/ARB	—	—	—	—	55	60	—	—	96	92	—	—	72.2	71.2	88	86
Beta-blocker	—	—	—	—	62	71	—	—	93	92	89.4	88.3	61.3	62.9	81	85

ACEI: angiotensin-converting enzyme inhibitor; ARB: angiotensin receptor blocker. ^1^Aspirin and/or clopidogrel. ^2^As per authors, all patients received aspirin at the time of primary PCI, but the percentage of patients that remained on aspirin in each arm is not stated.

**Table 3:** Safety outcomes.

Outcome	Studies	Participants	Statistical method	Effect estimate
GI adverse reactions	6	11575	Odds ratio (Peto, fixed, 95% CI)	1.06 [0.94, 1.20]
Treatment discontinuation	6	11575	Odds ratio (Peto, fixed, 95% CI)	1.11 [0.96, 1.28]
Infections	2	10198	Odds ratio (Peto, fixed, 95% CI)	1.04 [0.84, 1.28]
Myalgia	4	4683	Odds ratio (Peto, fixed, 95% CI)	1.16 [0.99, 1.36]
Cancer	2	10198	Odds ratio *R* (Peto, fixed, 95% CI)	0.97 [0.78, 1.21]
Myelotoxicity	6	11307	Odds ratio OR (Peto, fixed, 95% CI)	1.01 [0.56, 1.80]

## Data Availability

All data are referenced in the manuscript.

## Supplementary Materials

Supplementary Materials Figure S1: search strategy. Figure S2: risk of bias assessment of randomized trials. Figure S3: sensitivity analysis: primary outcome of MACE excluding trials with open-label design. Figure S4: risk of publication bias across studies. Figure S5: exploration of heterogeneity-exclusion of open-label trials. Table S1: GRADE quality assessment and summary of findings.
