# Supplementary material for: Meta-Analysis of Randomized Trials: Efficacy and Safety of Colchicine for Secondary Prevention of Cardiovascular Disease
Source: J Interv Cardiol. 2024 Mar 12;2024:8646351. doi: 10.1155/2024/8646351 (PMC10950412; doi:10.1155/2024/8646351)
Supplement: Supplementary Materials — Figure S1: search strategy. Figure S2: risk of bias assessment of randomized trials. Figure S3: sensitivity analysis: primary outcome of MACE excluding trials with open-label design. Figure S4: risk of publication bias across studies. Figure S5: exploration of heterogeneity-exclusion of open-label trials. Table S1: GRADE quality assessment and summary of findings. [file 8646351.f1.zip › Figure S1.docx]

**Figure S1: Search Strategy**

Databases: Embase, Ovid Healthstar, Ovid MEDLINE(R) In-Process & Other Non-Indexed Citations, Ovid MEDLINE(R) Daily and Ovid MEDLINE(R)

Search Strategy

1. exp colchicine/ or colchicine.mp. (44763)
2. exp Myocardial Ischemia/ (787240)
3. exp Cardiovascular Surgical Procedures/ (1142170)
4. exp Cardiovascular Diseases/ (6791445)
5. exp Stroke/ (308613)
6. exp Death/ or exp Death, Sudden, Cardiac/ or exp Death, Sudden/ (769542)
7. 2 or 3 or 4 or 5 or 6 (7644650)
8. 1 and 7 (6220)
9. limit 8 to humans (5601)
10. limit 9 to English language (4896)
11. limit 10 to (clinical trial, all or clinical trial, phase i or clinical trial, phase ii or clinical trial, phase iii or clinical trial, phase iv or clinical trial or comparative study or controlled clinical trial or multicenter study or randomized controlled trial or clinical study or observational study or pragmatic clinical trial) [Limit not valid in Embase,HealthSTAR; records were retained] (658)
