# Supplementary material for: Meta-Analysis of Randomized Trials: Efficacy and Safety of Colchicine for Secondary Prevention of Cardiovascular Disease
Source: J Interv Cardiol. 2024 Mar 12;2024:8646351. doi: 10.1155/2024/8646351 (PMC10950412; doi:10.1155/2024/8646351)

**Figure S2: Risk of Bias Assessment of Randomized Trials**

**COLCOT 2019**


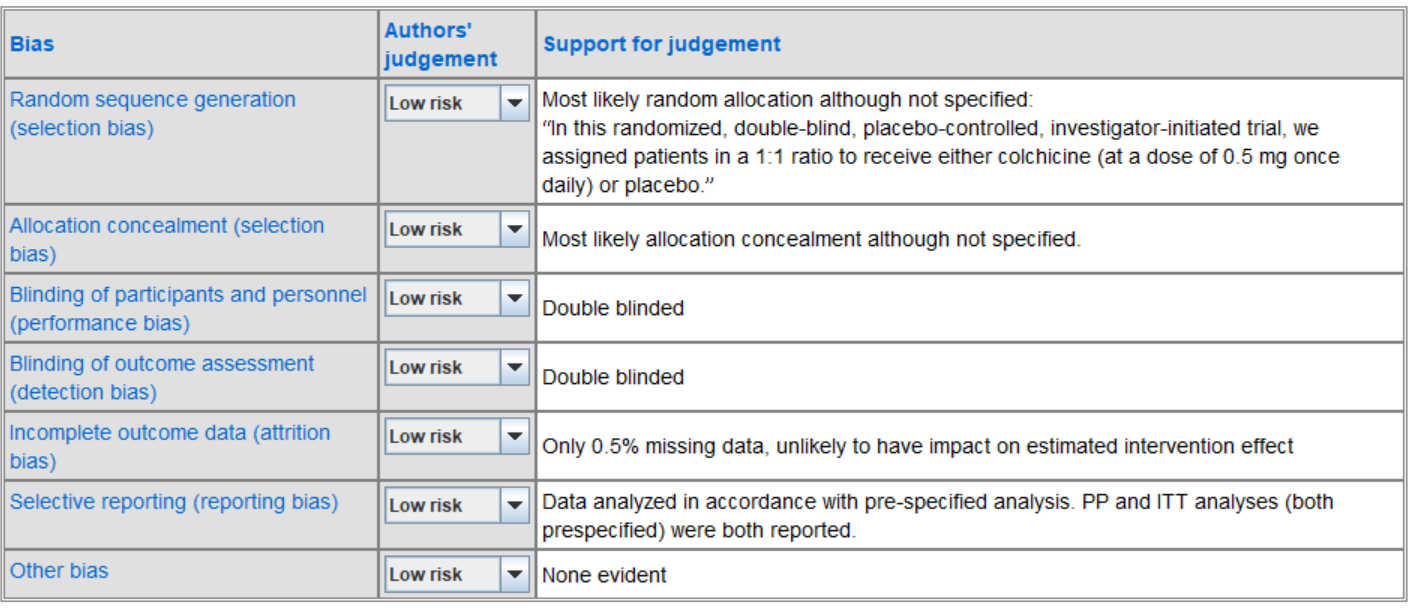


**COPS 2020**


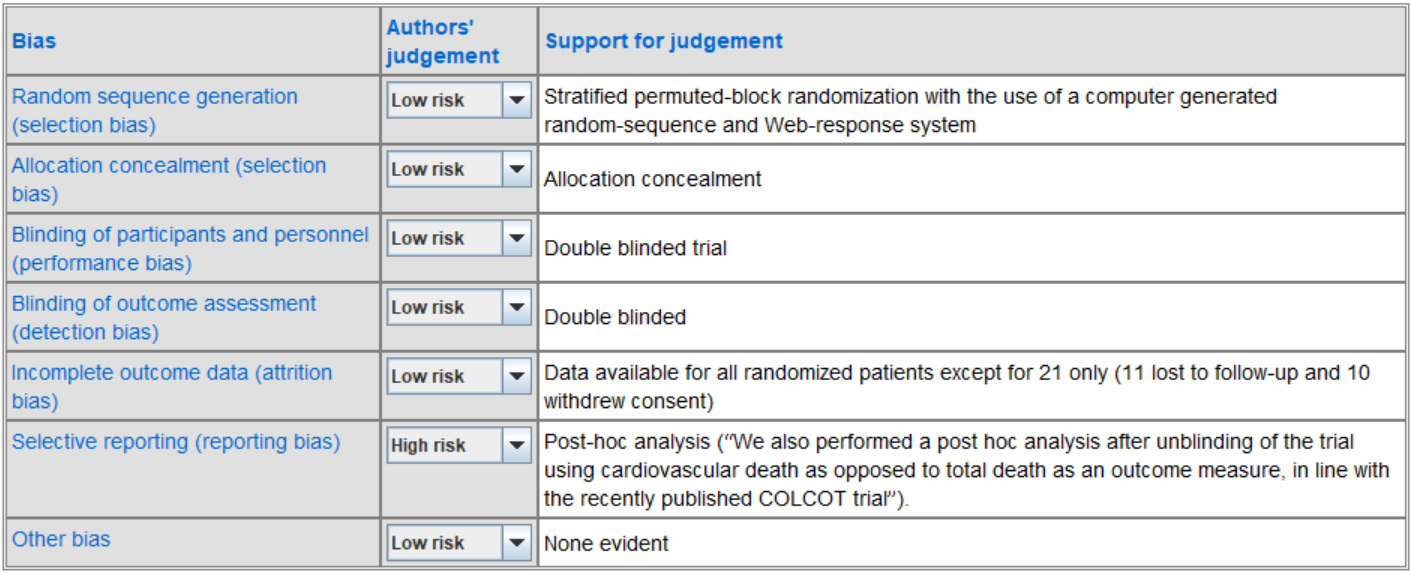


**Deftereos 2013**


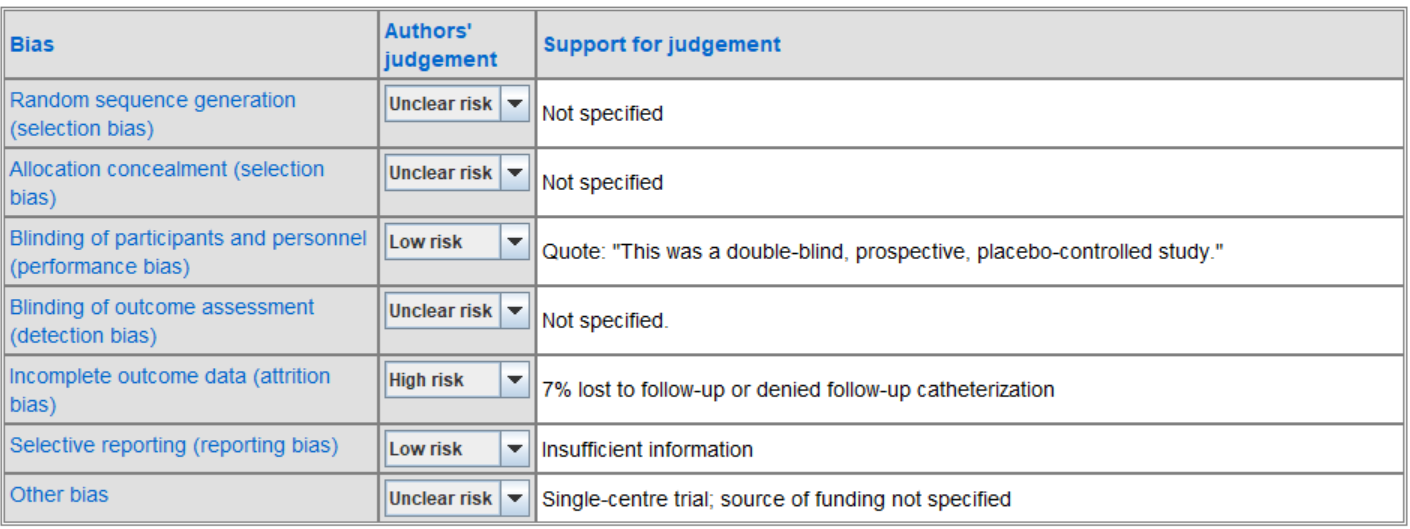


**LoDoCo 2013**


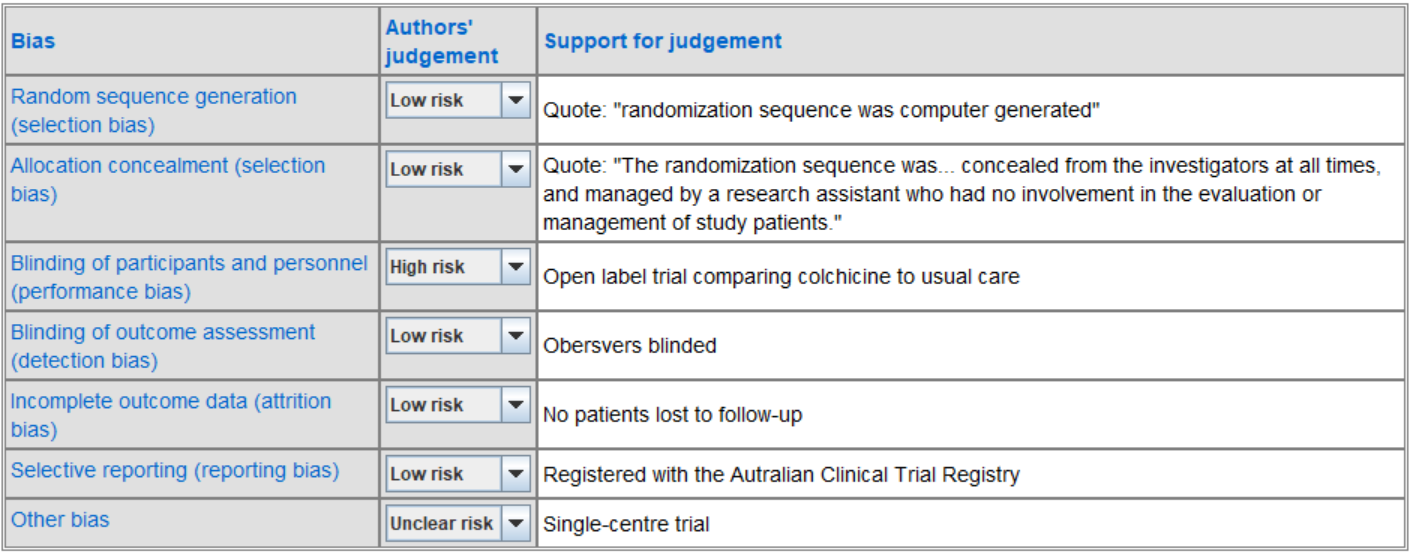


**LoDoCo-MI 2019**


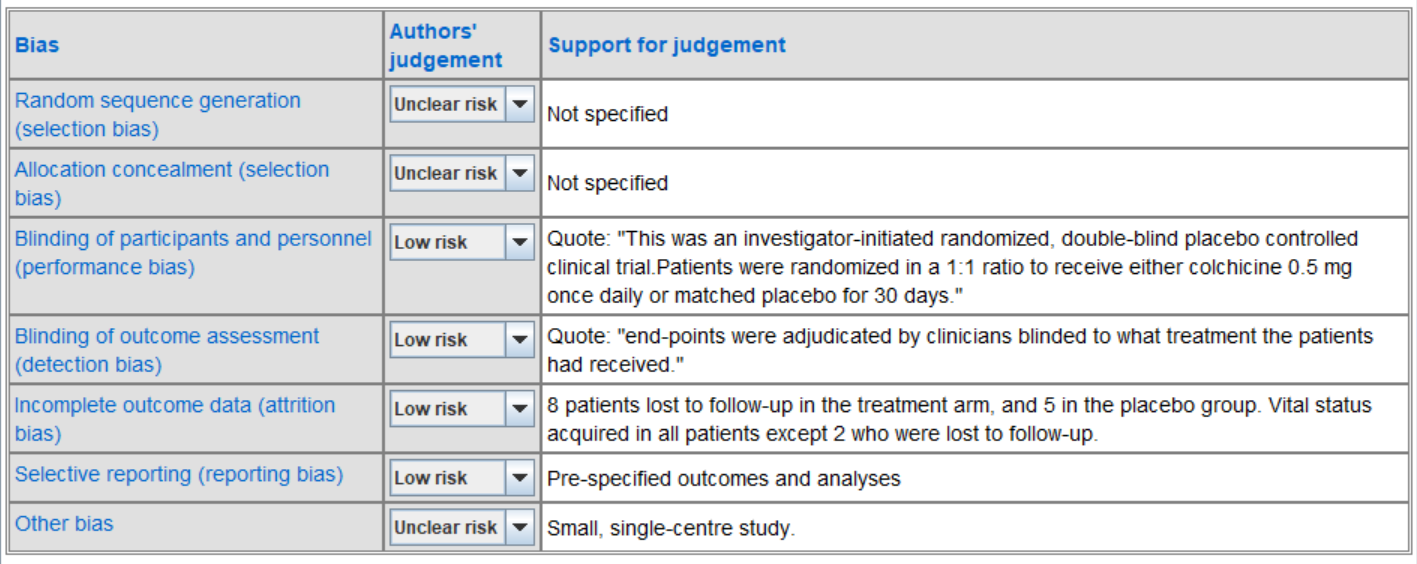


**LoDoCo 2020**


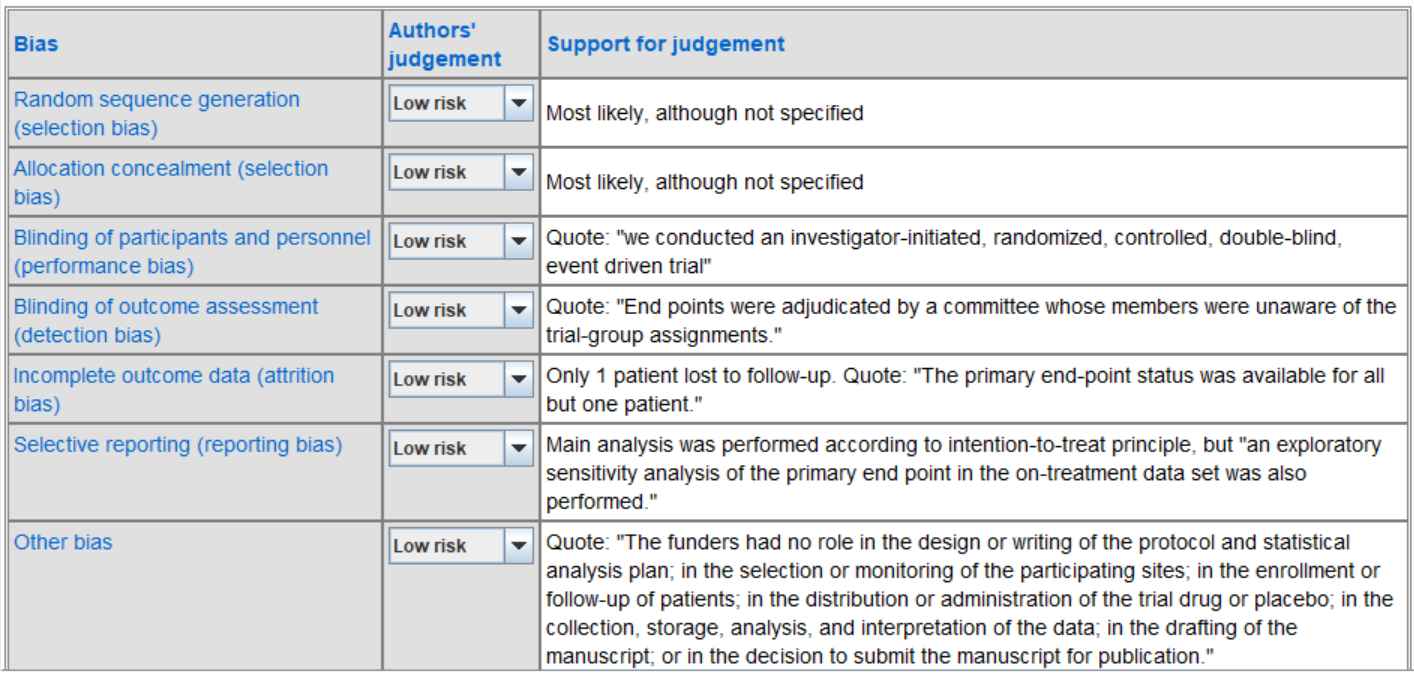


**Raju 2012**


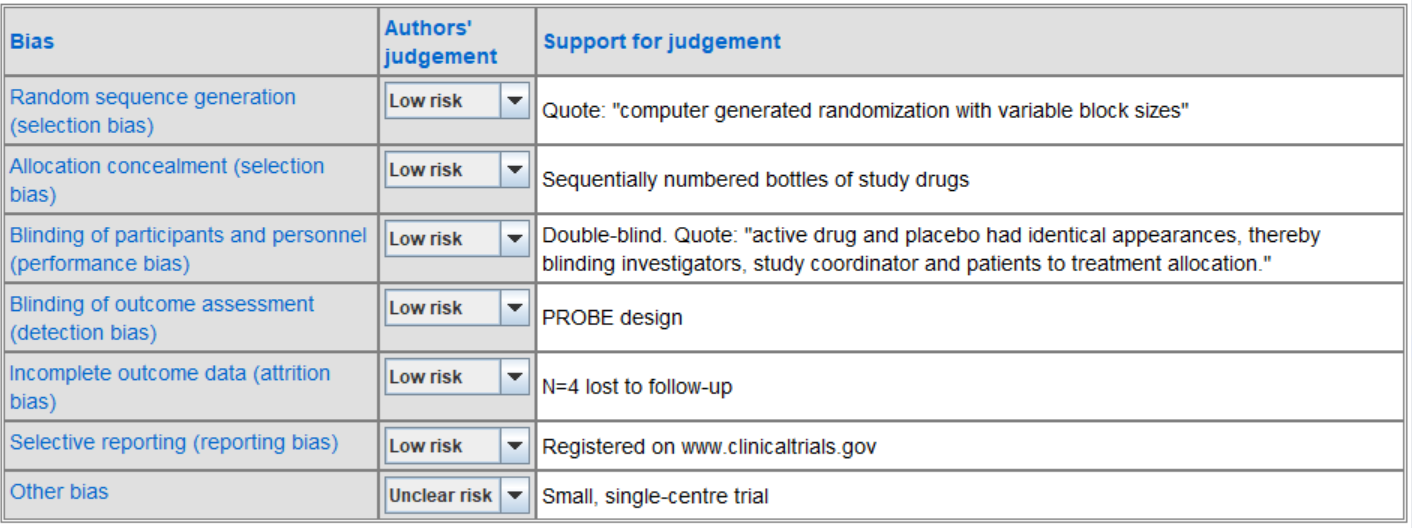


**COLIN 2017**


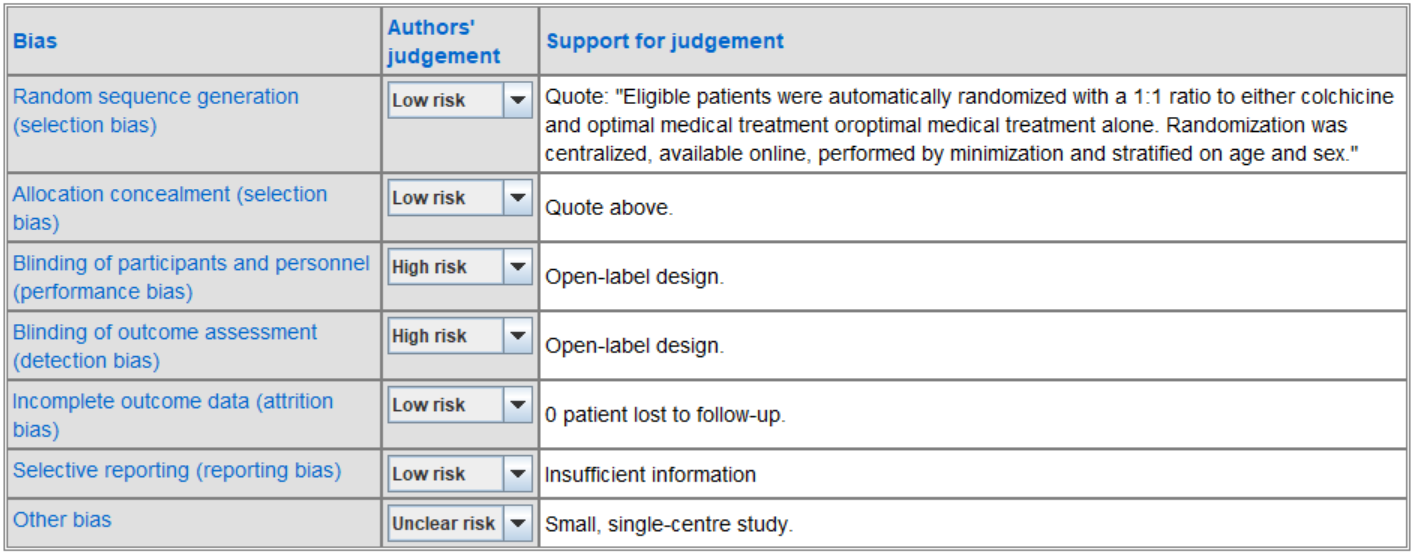

Supplement: Supplementary Materials — Figure S1: search strategy. Figure S2: risk of bias assessment of randomized trials. Figure S3: sensitivity analysis: primary outcome of MACE excluding trials with open-label design. Figure S4: risk of publication bias across studies. Figure S5: exploration of heterogeneity-exclusion of open-label trials. Table S1: GRADE quality assessment and summary of findings. [file 8646351.f1.zip › Figure S2.docx]
