# Supplementary material for: Meta-Analysis of Randomized Trials: Efficacy and Safety of Colchicine for Secondary Prevention of Cardiovascular Disease
Source: J Interv Cardiol. 2024 Mar 12;2024:8646351. doi: 10.1155/2024/8646351 (PMC10950412; doi:10.1155/2024/8646351)
Supplement: Supplementary Materials — Figure S1: search strategy. Figure S2: risk of bias assessment of randomized trials. Figure S3: sensitivity analysis: primary outcome of MACE excluding trials with open-label design. Figure S4: risk of publication bias across studies. Figure S5: exploration of heterogeneity-exclusion of open-label trials. Table S1: GRADE quality assessment and summary of findings. [file 8646351.f1.zip › Table S1.docx]

**Table S1: GRADE Quality Assessment and Summary of Findings**

| **Certainty assessment** | | | | | | | **№ of patients** | | **Effect** | | **Certainty** | **Importance** |
| --- | --- | --- | --- | --- | --- | --- | --- | --- | --- | --- | --- | --- |
| **№ of studies** | **Study design** | **Risk of bias** | **Inconsistency** | **Indirectness** | **Imprecision** | **Other considerations** | **CV mortality** | **placebo** | **Relative (95% CI)** | **Absolute (95% CI)** |  |  |
| **MACE** | | | | | | | | | | | | |
| 8 | randomised trials | not serious | not serious | not serious ^a^ | not serious | none ^b^ | 365/6088 (6.0%) | 526/6063 (8.7%) | **RR 0.67** (0.56 to 0.80) | **29 fewer per 1,000** (from 38 fewer to 17 fewer) | ⨁⨁⨁⨁ HIGH | CRITICAL |
| **All-cause mortality** | | | | | | | | | | | | |
| 8 | randomised trials | not serious | very serious ^c^ | not serious ^a^ | serious ^d^ | none ^b^ | 129/6088 (2.1%) | 116/6063 (1.9%) | **RR 1.04** (0.64 to 1.69) | **1 more per 1,000** (from 7 fewer to 13 more) | ⨁◯◯◯ VERY LOW | CRITICAL |
| **Cardiovascular mortality** | | | | | | | | | | | | |
| 8 | randomised trials | not serious | serious ^e^ | not serious ^a^ | serious ^d^ | none ^b^ | 44/6088 (0.7%) | 56/6063 (0.9%) | **RR 0.82** (0.55 to 1.22) | **2 fewer per 1,000** (from 4 fewer to 2 more) | ⨁⨁◯◯ LOW | CRITICAL |
| **Myocardial infarction** | | | | | | | | | | | | |
| 6 | randomised trials | not serious | not serious | not serious ^a^ | not serious | none ^b^ | 185/5948 (3.1%) | 244/5927 (4.1%) | **RR 0.74** (0.57 to 0.95) | **11 fewer per 1,000** (from 18 fewer to 2 fewer) | ⨁⨁⨁⨁ HIGH | CRITICAL |
| **Coronary revascularization** | | | | | | | | | | | | |
| 4 | randomised trials | not serious | not serious | not serious ^a^ | not serious | none ^b^ | 163/5524 (3.0%) | 239/5538 (4.3%) | **RR 0.58** (0.37 to 0.92) | **18 fewer per 1,000** (from 27 fewer to 3 fewer) | ⨁⨁⨁⨁ HIGH | IMPORTANT |
| **Stroke** | | | | | | | | | | | | |
| 8 | randomised trials | not serious | not serious | not serious ^a^ | serious ^d^ | none ^b^ | 25/6088 (0.4%) | 54/6063 (0.9%) | **RR 0.49** (0.30 to 0.78) | **5 fewer per 1,000** (from 6 fewer to 2 fewer) | ⨁⨁⨁◯ MODERATE | IMPORTANT |
| **GI adverse events** | | | | | | | | | | | | |
| 6 | randomised trials | not serious | not serious | not serious ^a^ | not serious | none ^b^ | 594/5783 (10.3%) | 567/5792 (9.8%) | **RR 1.15** (0.94 to 1.40) | **15 more per 1,000** (from 6 fewer to 39 more) | ⨁⨁⨁⨁ HIGH | IMPORTANT |

**CI:** Confidence interval; **RR:** Risk ratio

#### Explanations

a. We judged this as “not serious” because the two included trials directly compared the intervention we are interested in (colchicine) versus placebo or usual care (the comparator we are interested in); the intervention is delivered to populations we prespecified.

b. While we are not completely confident that publication bias is absent and there are not enough studies to do a funnel plot, our search criteria covered multiple sources including MEDLINE, EMBASE and the Cochrane databases, trial registries (clinicaltrials.gov), and reference lists from full text manuscripts and published systematic reviews.

c. Two trials showed potential increase in all-cause mortality with colchicine (COPS and LoDoCo2).

d. Downgraded due to small number of events

e. One trial showed potentially increased cardiovascular mortality with colchicine therapy (COPS)
